# Whole-genome sequencing of *Histophilus somni* strains isolated in Russia

**DOI:** 10.14202/vetworld.2023.272-280

**Published:** 2023-02-14

**Authors:** Svetlana Yatsentyuk, Julia Pobolelova, Veronika Gordeeva, Irina Timofeeva

**Affiliations:** Department of Biotechnology, Russian State Center for Animal Feed and Drug Standardization and Quality, 5, Zvenigorodskoe Highway, Moscow, Russia

**Keywords:** antibiotic resistance genes, *Histophilus somni*, next-generation sequencing, virulence factors

## Abstract

**Background and Aim:**

*Histophilus somni* is a Gram-negative bacterium belonging to the *Pasteurellaceae* family that can cause bovine histophilosis. *Histophilus* may act as a commensal or opportunistic bacterial cattle pathogen. Comparing genomes of the pathogenic strain 2336 with the non-pathogenic preputial 129Pt isolate revealed some putative virulence factors. The study of the complete genomes of *H. somni* strains circulating in Russia has never been conducted before. This study aimed to identify genetic features of the *H. somni* strains isolated in Russia and evaluate the possibility of using strains for vaccine development.

**Materials and Methods:**

Three strains of *H. somni* were isolated from different sources. Strain 188-VIEV was isolated from a vaginal swab sample of cattle with endometritis. 532-VIEV and 551-VIEV were cultured from the cryopreserved bull semen samples imported from Canada. *Histophilus somni* strain ATCC 700025 provided by ATCC (American Type Culture Collection) was also used in the study. DNA extraction was performed using QIAamp DNA Mini Kit (QIAGEN, USA). The whole-genome sequencing of the four strains was performed using Illumina Miseq. The comparison of the resulting sequences with the complete genomes of *H. somni* 2336 and 129Pt, and detection of the resistance genes and virulence factors, was performed using the ResFinder and Virulence Factor Database web services.

**Results:**

The genome size of the samples varied from 1.9 to 2.3 Mb. The number of coding sequences varied from 1795 to 2256. The average sequence density was 90%. The total guanine-cytosine (GC) content was 36.8%–37.2%, which coincided with data previously obtained for *H. somni*. Three out of four studied strains encoded putative virulence factors such as filamentous hemagglutinin homologs, lipooligosaccharide biosynthesis proteins, and proteins involved in iron transport and utilization. The Ser83Ile substitution was identified in the DNA topoisomerase II (*gyrA*) in *H. somni* strains 532-VIEV and 551-VIEV cultured from bull semen which led to resistance to fluoroquinolones. The gene (*AAC-6-Ia + APH-2*’’) encoding a bifunctional aminoglycoside modification enzyme was detected in strain 551-VIEV.

**Conclusion:**

Strains with virulence genes identified could be candidates for designing vaccines and potentially represent antigen sources. The results show that antibiotic-resistant *H. somni* can be spread with semen used for artificial insemination.

## Introduction

*Histophilus somni* is a Gram-negative, non-spore-forming, non-capsulated, non-motile, pleomorphic bacterium belonging to the phylum Proteobacteria, class Gammaproteobacteria, family Pasteurellaceae. *Histophilus somni* was proposed as a common name for the three species *Haemophilus somnus*, *Haemophilus agni*, and *Histophilus ovis* [1]. Originally described as a “Haemophilus-like” organism, *H. somni* was first isolated in 1956 in the USA from cattle with meningoencephalitis [1]. *Histophilus somni* normally lives on the bovine mucous membranes and can be described as an opportunistic pathogen. *Histophilus* *somni* can cause various diseases, including respiratory and reproductive diseases, myocarditis, polyarthritis, mastitis, eye diseases, and sepsis. However, some *H. somni* isolates may be entirely non-pathogenic. Although *H. somni* no longer relates to the genus *Haemophilus*, many of the genes responsible for the virulence of the type species of the genus - *Haemophilus influenzae*, have been retained in *H. somni*. Some important human pathogens (e.g., *Bordetella, Neisseria*, and *Haemophilus* spp.) and *H. somni* share several features such as capability to form biofilms and display lipopolysaccharide phase variation and modification with sialic acid and phosphorylcholine (ChoP). Thus, *H. somni* may prove to be a useful model in host-pathogen interaction studies.

**Figure-1:**
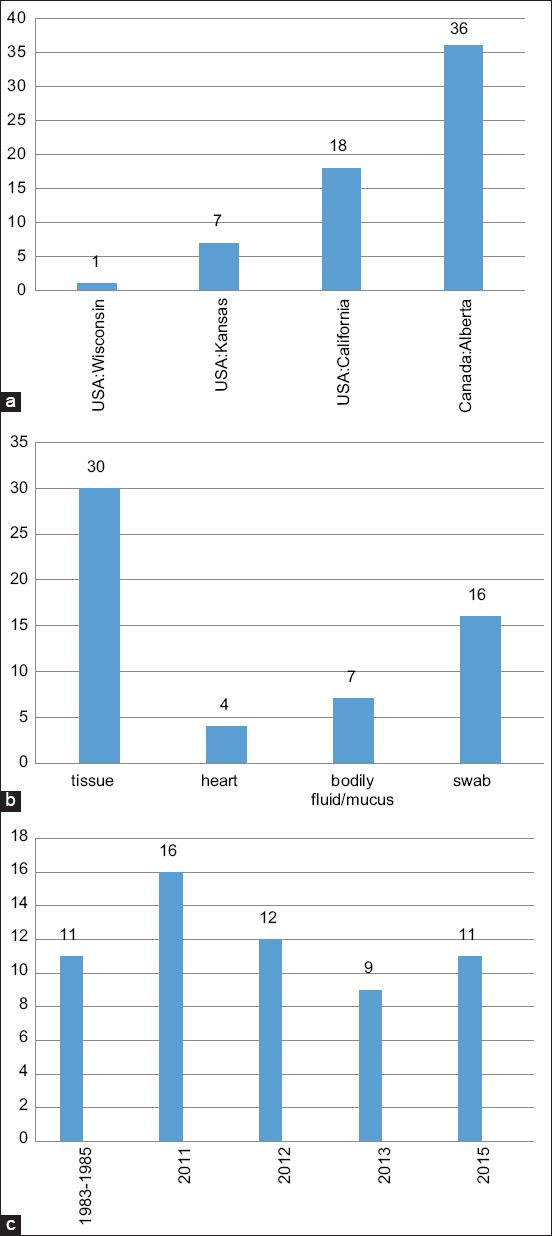
Overview of the *Histophilus somni* complete genomes in National Center for Biotechnology Information (a) country, (b) isolation source, (c) date of isolation.

Whole-genome sequencing of bacterial genomes opens up new opportunities for further clarification of the taxonomic relationships between species and identification of potential genetic determinants of pathogenicity. The information obtained will allow scientists to select strains to develop new vaccines. In 2007, the first complete genome sequence of the *H. somni* 129Pt strain, isolated from bull pre­putium and characterized as non-pathogenic [2]. Then, the genome sequence of the *H. somni* 2336 strain isolated from lungs of a calf with pneumonia was determined [3]. At present, information regarding the genetic structure of circulating strains is being collected and reviewed. The National Center for Biotechnology Information (NCBI) collection contains about 60 genome sequences of *H. somni* isolated from cattle, 29 of which are complete sequences, and 31 are draft. All sequences were provided by the USA (Kansas, California, Wisconsin) and Canada (Alberta) (Figure-1).

This study aimed to obtain information regarding genome characteristics of the *H. somni* strains isolated in Russia and assess the possibility of using them for vaccine development.

## Materials and Methods

### Ethical approval

This study analyzed the complete genomes of the *H. somni* strains; therefore, ethical approval is not required.

### Study period and location

This study was conducted from March 2021 to August 2022 at the Russian State Center for Animal Feed and Drug Standardization and Quality.

### Samples

*Histophilus* *somni* cultures used in this study were isolated from the vaginal swab sample collected from a cow with clinical signs of endometritis (188-VIEV), and cryopreserved bull semen samples imported from Canada and intended to be used for artificial insemination (532-VIEV and 551-VIEV). In addition, the strain of *H. somni* ATCC 700025 provided by the American Type Culture Collection was studied.

### Whole-genome sequencing

Genomic DNA was isolated from cultures using the QIAamp DNA Mini Kit (Qiagen, Germany). The DNA concentration was measured using Quantus Fluorimeter (Promega) and QuantiFluor^®^ONE dsDNA System Reagent Kit (Promega, USA). The library for sequencing was prepared according to the standard protocol. Tagmentation and indexing were performed according to the Illumina manual NexteraXT DNA Library Prep Kit. Reference Guide (#15031942) using NexteraXT Reagent Kit. Prepared libraries were denatured and diluted for sequencing according to the normalization procedure described in the Illumina Denature and Dilute Libraries Guide (#15039740). Sequencing using MiSeq Reagent Nano Kit v2 was performed in accordance with the MiSeq System Illumina Experiment Manager User Guide (#15031335v05).

### Genome assembly and comparative genomic analysis

Analysis of FASTQ files to evaluate the quality of nucleotide sequences was carried out using the FastQC_0.11.17 software (http://www.bioinformatics.babraham.ac.uk/projects/fastqc/). Adapter sequences were removed and low-quality reads were filtered usingTrimmomatic v.0.36 software (http://www.usadellab.org/cms/?page=trimmomatic) according to the following ILLUMINACLIP parameters: NexteraPE-PEfa:2:30:10, SLIDINGWINDOW: 4:20.

The *de novo* assembly of the bacterial genome was carried out with SPAdes_2.11.1 as well as implementing an error correction program and automated k-mer size selection. The main characteristics of the assembly were obtained using the QUAST 4.6.3 program. Various k-mers 21, 33, 55, 77, 99, and 127 were used for assembly. Contigs smaller than 500 bp and coverage value below 10x were excluded from further analysis. The remaining contigs were aligned against the *H. somni* 2336 genome (NCBI Reference Sequence: NC_010519.1) using the software MAUVE v.20150226. Genotyping was performed using a common k-mer based search method and the online KmerFinder tool hosted by the Center for Genomic Epidemiology of the Technical University of Denmark (version 2.1).

Identification of the main virulence factors and antibiotic resistance genes was conducted using The Virulence Factor Database (VFDB) (http://www.mgc.ac.cn/VFs/) and the RAST online server (https://rast.nmpdr.org/) [4].

The results of the whole-genome sequencing were compared with the genomes of pathogenic *H. somni* 2336 (NCBI Reference Sequence: NC_010519) and non-virulent 129Pt (NCBI Reference Sequence: CP000436).

To compare the studied genomes with pathogenic *H. somni* 2336 and non-pathogenic 129Pt, Venn diagrams were constructed based on clustering of amino acid sequences using parameters identity = 95% and cover = 80% without hypothetical proteins.

### Results and Discussion

Whole-genome sequencing of four *H. somni* cultures was carried out. The genome size of various strains ranged from 1.9 to 2.3 Mb. The number of coding sequences varied from 1795 to 2256 bp. The average coding sequence density was 90%. The overall guanine-cytosine (GC) content was from 36.8% to 37.2%. The general features of the *H. somni* samples are listed in Table-1. The sequences of the *H. somni* 188-VIEV, 532-VIEV, 551-VIEV, and *H. somni* ATCC 700025 have been deposited in the GenBank sequence database of the NCBI under accession numbers JAHSQM000000000.1, JAHSQL000000000.1, JAHSQK000000000.1, JAHSQI000000000.1. BioProject: PRJNA736593.

**Table-1:** Genome descriptions.

Strain	ATCC 700025	188-VIEV	551-VIEV	532-VIEV
Isolation source	No data	Vaginal swab	Semen	Semen
Isolation_ date		2018	2018	2018
Number of coding sequences	2037	2256	1948	1795
Overall coding density	90.6%	90.9%	90.1%	89,9%
Size (bp)	2 126 663	2 290 854	2 036 892	1 906 694
Number of subsystems	249	260	246	235
GC- content	37.2	36.9	37.1	36.8
5S ribosomal genes	4	4	4	4
16S ribosomal genes	1	2	2	1
23S ribosomal genes	1	3	1	1
Number of tRNA genes	39	56	44	42
Prophage region	2	1	1	1

GC=Guanine-cytosine

Novel *H. somni* genomes were compared with the pathogenic 2336 strain genome and the non-pathogenic 129Pt. The genome size of the *H*. *somni* strain 2336 was found to be larger than the genome of strain 129Pt. It is assumed that the larger genome size is associated with the presence of virulence factor genes [5]. The genome sizes of studied samples 551-VIEV and 532-VIEV isolated from the bull semen samples were similar to the genome size of the non-virulent strain 129Pt. The genome sizes of *H. somni* strains ATCC 700025 and 188-VIEV were 2.127 Mb and 2.290 Mb, respectively, which were similar to the genome size of 2336 strain isolated from a diseased animal. Furthermore, the number of coding sequences in the studied strains was different. Samples isolated from the bull semen contained <2000 genes, and the other two samples were more than 2000 genes in size (Table-1).

**Figure-2:**
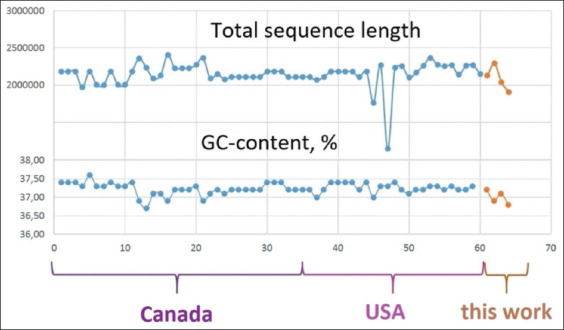
Comparison of total sequence length and guanine-cytosine-content of the *Histophilus somni* sequences deposited in the GenBank database.

**Figure-3:**
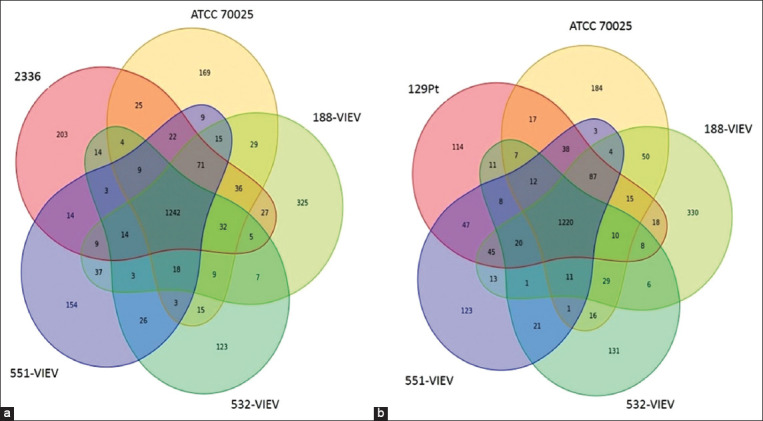
Sequence comparison of *Histophilus somni* strains ATCC 700025, 188-VIEV, 551-VIEV, and 532-VIEV with the reference sequence of (a) pathogenic strain 2336 (b) non-pathogenic 129Pt.

Despite the difference in the overall size of the genomes, the GC content of the *H. somni* sequences in the NCBI database was approximately the same (36.6%–37.6%), and we observed a similar result in the studied strains (Figure-2). A comparison of the genomes is shown in Figure-3.

When comparing studied genomes with the pathogenic strain *H. somni* 2336, it was found that 1242 genes are common to all samples. The *Histophilus* 188-VIEV genome had the highest number of unique genes (325 out of 2256, i.e., 14.40%), while the genomes of the remaining three samples had approximately the same number of unique genes (ATCC 700025 - 8.3%, 551-VIEV - 7. 9%, and 532-VIEV - 6.85%). The number of genes present in the four studied strains, except for 532-VIEV, was the highest (71). It was found that in other combinations, the number of genes common to studied *H. somni* strains was much lower. Nine genes were identified in all strains, except for 188-VIEV. Fourteen genes were identified in all strains, except for ATCC 700025, and 32 genes were identified in all strains, except for 551-VIEV. Similar results were obtained while comparing studied strains with the non-pathogenic *H. somni* 129Pt. There were 1220 common genes. 188-VIEV strain contained even a higher number of unique genes (330 out of 2256, i.e., 14.63%). The number of common genes for all studied strains, except for 532-VIEV, was also the largest - 87.

To determine the potential pathogenicity of the studied *Histophilus* strains, and to select candidate strains for vaccine development, a search for virulence factors was conducted. According to the previously published reports, the studies showed that several genes were present in the pathogenic 2336 strain and were absent in the non-pathogenic 129Pt strain [5]. The study of some of these genes revealed a possible mechanism of *H. somni* pathogenicity. However, the role of most *H. somni* genes in virulence is only suspected. The results of the search for the main virulence factors in the studied *H. somni* sequences are summarized in Table-2.

**Table-2:** Genes, associated with virulence and VNTR detected in studied *H. somni* samples.

Gene	Protein annotation	*H. somni* strains					

		2336	129Pt	ATCC 700025	188-VIEV	551-VIEV	532-VIEV
Filamentous hemagglutinins							
*ibpA*	Immunoglobulin binding protein A	+	-	+	+	-	+
*ibpB*	Immunoglobulin binding protein B	+	-	+	+	-	+
Iron transport and utilization							
*tbpA*	Transferrin-binding protein A	+	+	+	+	+/-	+
*tbpB*	Transferrin-binding protein B	+	+	+	+	+/-	+
*fur*	Ferric uptake regulation protein	+	+	-	-	-	-
LOS							
*lob1*	Galactosyl Transferase	+ (33 CAAT)	+ (36 CAAT)	+ (18 CAAT)	+ (26 CAAT)	+/-	+ (18 CAAT)
*lob2A*	Glycosyl transferase	+	+	+	+	-	+
*lob2B*	N-acetylglucosamine transferase	+ (22 GA)	-	+ (25 GA)	+	-	+ (24 GA)
*lob2C*	Glycosyltransferase-like protein	+	+	+	+	+	+
*lob2D*	Glycosyltransferase-like protein	+/- (18 CAGT)	+ (29 CAGT)	+ (18 CAGT)	+ (23 CAGT)	+/- (22 CAGT)	+ (18 CAGT)
*licA*	Choline kinase	+ (25 AACC)	+ (41 AACC) IS1016	+ n/a	+ (24 AACC)	+ n/a	+ (32 AACC)
*licB*	Choline transporter	+	+	+	+	+	+
*licC*	CTP: Phosphocholine cytidylyltransferase	+	+	+	+	+	+
*licD*	LOS choline phosphotransferase	+	+	+	+	+	+
*neuA*	CMP-N-acetyl-5-neuraminic acid synthetase	+	+/-	+	+	+/-	+
*siaA*	Sialyltransferase	+	+/-	+	+	+/-	+
*lsgB*	CMP-N-acetylneuraminate-beta- galactosamide- alpha-2,3- sialyltransferase	+	-	+/-	+/-	-	-

LOS=Lipooligosaccharides, +=Full gene is present; -=No copies of the gene are present; +/-=Gene is truncated; n/a=No VNTR data, VNTR=Variable number tandem repeats, CMP=Cytidine 5’-monophosphate

### Iron transport and utilization

One of the mechanisms of iron utilization required by bacterial pathogens is the direct binding of host transferrin by bacterial surface receptors [6]. The functioning of this mechanism is provided by two outer membrane proteins - transferrin-binding proteins A and B, encoded by the *tbpA* and *tbpB* genes, respectively. Transferrin-binding proteins of most microorganisms usually bind only one type of transferrin, but proteins of *Mannheimia haemolytica*, *P. multocida*, and *H. agni* are capable of binding ovine, bovine and goat transferrins, but not transferrin of non-ruminant hosts. *Histophilus somni* receptors bind bovine transferrin, which in part accounts for *H. somni* specificity to cattle [7]. Recent studies have found that *H. somni* is also capable of binding sheep and goat transferrins [6]. Analysis of the complete genomes of *H. somni*, provided in the GenBank database, revealed that *tbpA* and *tbpB* were present in all sequences. Genes *tbpA* and *tbpB* were determined in 3 out of 4 studied *H. somni* samples. It was found that the *tbpA* gene was truncated in 551-VIEV sample, while the *tbpB* gene was not detected. Nucleotide sequence alignment showed that the *tbpA* gene was represented by several regions; in particular, the 4190–4650 region was highly (100% identity) conserved in all the studied samples. Some other *tbpA* regions were 100% identical in the genomes of strains 2336, 129Pt and ATCC 700025, although differences were identified in strains 188-VIEV, 532-VIEV, and 551-VIEV. The *tbpA* sequence identity observed in these samples ranged between 88.24% and 99.14%. The nucleotide sequences of *tbpB* genes were found to be more variable: The identity of *tbpB* genes in studied samples and strain 2336 was 70%.

The transferrin-binding protein genes are generally repressed by iron [8]. For many bacteria, this is associated with the ferric uptake regulator (Fur) protein that acts to repress the expression of *tbp* genes in the presence of iron. Fur gene homologs and Fur-binding motif (AATTATTATC) upstream of the genes involved in iron utilization have been observed in *H. somni* 129Pt, *H. influenzae*, *H. ducreyi* sequences [9, 10]. However, we did not identify Fur motif upstream of the *tbpA* and *tbpB* genes in the genome of the studied isolates.

### Filamentous hemagglutinins

Genes of the immunoglobulin-binding proteins *ibpA* and *ibpB* may play an important role in *H. somni* virulence [5]. Studies have shown that *H. somni* strains from asymptomatic carriers lacked the *ibpA* gene [11], and the strains were susceptible to bovine serum [11, 12]. In contrast, strains isolated from diseased animals were serum-resistant and contained *IbpA* [5, 12].

It has been previously reported that *ibpA* contains two large DR1/DR2 direct repeats near the C-terminus [11, 13, 14]. In addition, it was found that one DR was enough for the *H. somni* virulence [7, 15]. It was found that the Fic motif in the DR1/DR2 domains of the IbpA protein causes cytotoxicity of *H. somni* for epithelial and phagocytic cells, which may interfere with the bactericidal activity of these cells [14]. The sequences of *H*. *somni* strains ATCC 700025, 188-VIEV, and 532-VIEV have both the complete *ibpA* gene and DR1/DR2 direct repeats, which may indicate the potential virulence of these strains. In the genome sequences deposited in the GenBank database, the *ibpA* gene was found in *H. somni* samples isolated in Canada from bovine tissues (NCBI Reference Sequences: CP066558-CP066567). It is most likely that these isolates were pathogenic. At the same time, the *IbpA* gene was not observed in *H. somni* samples collected in the USA from swabs of the respiratory tract of cattle [16].

**Figure-4:**
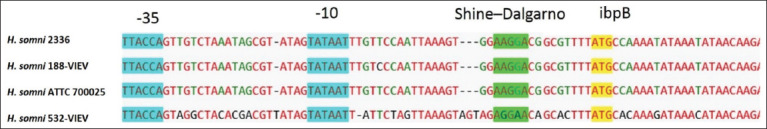
*Histophilus somni* *IbpB* promoter and the Shine-Dalgarno sequence motif alignment.

The *ibpB* gene is usually located in front of the *ibpA* gene. *Ibp* genes were not observed in both 551-VIEV and 129Pt strains. This indicates the potential non-pathogenicity of 551-VIEV. The sequence of the *ibpB* in *H. somni* 188-VIEV is 99.32% identical to the sequence of the pathogenic strain 2336. The identity value of *ibpB* gene in 532-VIEV and 2336 was significantly lower and corresponded to 96.32%. A mutation in the *ibpB* Shine-Dalgarno sequence motif was found in 532-VIEV. The presence of this mutation will likely affect the translation efficiency. However, the promoter sequences 10 Pribnow-box (TATAAT; 32 bp) and −35 (TTACCA; 63 bp) in 532-VIEV coincide with the other studied *H. somni* *ibpB* sequences (Figure-4). We also searched for promoters and the Shine-Dalgarno sequence motif of the *ibpA* gene. Sequence analysis did not reveal any substitution in the studied samples.

### Lipooligosaccharides (LOS)

The *Histophilus* LOS are large molecules with multiple functions. Lipooligosaccharides can provide protection to *H. somni* against host defenses and may act as an adhesin or endotoxin. Lipooligosaccharides are capable of undergoing phase changes in retaliation for increased host immune responses. Phosphorylcholine (ChoP) can be a part of LOS and promote adhesion to epithelial cells and is also capable of undergoing phase changes [7].

The major outer core proteins of the *H. somni* LOS are products of the *lob* gene families (*lob1, lob2A, lob2B, lob2C*, and *lob2D*) and *lic* (*licA, licB, licC*, and *licD*) [17]. Notably, all the recently tested non-pathogenic *H. somni* isolates, which have been isolated from the bovine prepuce, are either incapable of undergoing LOS phase variations, or the speed of the LOS phase variations can be very slow [18]. This may be due to the absence of a functional glycosyltransferase gene, or to the influence on the expression of a certain number of variable number tandem repeats (VNTR).

The *licA* sequence of the pathogenic *H. somni* strain 2336 contains 25 repeats of the tetranucleotide unit 5’-AACC-3’ located before one of the three potential start codons. The effect of the number of tandem repeats on ChoP expression was analyzed and it was shown that the presence of 24, 27, or 43 repeats did not result in a frameshift, and the expression of a functional product could be expected [17]. When the number of repeats was 23, 29, or 42, the expression was absent. It has also been observed that the absence of VNTR does not affect ChoP expression [17]. Analysis of the *licA* sequences of our samples showed that 24 tandem repeats were present in the 188-VIEV sample; therefore, ChoP expression was potentially possible. As to 532-VIEV sample, 32 VNTRs were identified, and it was difficult to predict the possibility of expression using the available data. The contigs obtained in strains ATCC 700025 and 551-VIEV did not allow us to determine the amount of VNTR.

The previous studies revealed the absence of ChoP in the LOS of the *H. somni* 129Pt. Furthermore, the mobile element IS1016 was detected in the *licA* of this strain [2]. A search for IS1016 revealed a truncated IS1016 sequence (516 bp) in the 551-VIEV sample. It was not adjacent to any gene and was the only sequence in the contig. Two copies of IS1016 were found in 532-VIEV - ~650 bp, and 387 bp. These copies were located within the hypothetical protein and did not appear to impact the expression of important genes. IS1016 was detected before putative heme iron utilization protein in the strain ATCC 700025 sequence. Sequences *licA, licB, licC*, and *licD* were identified in all analyzed samples.

### Decreased activity of host immune response

The outer core oligosaccharide structure of the LOS of some strains of *H. somni* mimics that of the host glycosphingolipids to protect against an immune response.

A type of antigenic mimicry is the incorporation of N-acetyl-5-neuraminic acid (Neu5Ac, or sialic acid), which is a common surface component of eukaryotic cells, onto the surface of a bacterial cell. It has been shown that the *neuA* gene is responsible for the synthesis of this acid; it was found in all the sequences we studied. However, it was found that the *neuA* gene in the 551-VIEV sequence was truncated as in the 129Pt strain. The protein encoded by the *neuA* was 41 amino acids less in size compared to the sequences of other isolated strains.

Strain 2336 produces two sialyltransferases encoded by the *siaA* and *lsgB* genes, which allow sialylation of LOS regardless of phase variations [5]. The 129Pt lacks the *lsgB* gene, and the truncated *siaA* gene encoded 75 amino acids out of 297. As regards to other studied samples, the complete *siaA* gene is present in the ATCC 700025, 188 – VIEV, and 532-VIEV. Only part of *siaA* gene was detected in the sample 551-VIEV. The complete *lsgB* gene was not observed in 551-VIEV as well as in 129Pt strain. Truncated variants of the *lsgB* gene were present in the ATCC 700025 and 188-VIEV samples, which indicates that LOS can be sialylated in the ATCC 700025, 188-VIEV, 532-VIEV samples to avoid the host defenses.

The analysis of the genomes of the *H. somni* strains isolated in Canada from animal tissues and presumably pathogenic (NCBI Reference Sequences: CP066558-CP066567, CP042983-CP043001) showed that they have complete *siaA* and *lsgB* genes.

### Antibiotic resistance

The problem of *H. somni* antibiotic resistance has been widely discussed in recent years [19–21]. In 2021, the phenotypic antibiotic resistance of the *H. somni* samples 188- VIEV, 532- VIEV, and 551-VIEV isolated in Russia was studied [22]. The results of the study of aminoglycosides resistance were the most interesting ones. In our study, we found that the 551-VIEV was phenotypically resistant to the aminoglycosides - kanamycin, neomycin, and streptomycin. The analysis of the 551-VIEV genome performed in this work revealed the presence of the *AAC-6-Ia* *+ APH-2*’’ gene encoding a bifunctional enzyme. This enzyme has both acetyltransferase and phosphotransferase activity. It has been observed that phosphotransferase induces resistance to kanamycin, gentamicin, and tobramycin, while acetyltransferase induces resistance to kanamycin, tobramycin, netilmicin, amikacin, and isepamycin [23]. Sequence analysis of 551-VIEV revealed that the *AAC(6)-IIa* region was highly conserved across different bacteria. The sample’s APH region was 100% identical to the APH region of *Enterococcus faecium* (NCBI Reference Sequence: WP_016439437.1). The APH of *H. somni* differs from the *E. coli* APH (NCBI Reference Sequence: WP_063840674.1) by single-point substitutions, while the *Campylobacter* APH (NCBI Reference Sequence: WP_057038954.1) has the biggest differences.

According to phenotypic data, 551-VIEV and 532-VIEV were also resistant to fluoroquinolones; ciprofloxacin, enrofloxacin, and norfloxacin. Samples 188-VIEV and ATCC 700025 were susceptible to fluoroquinolones. Therefore, in this study, we searched for genetic markers of fluoroquinolones resistance in the 551-VIEV and 532-VIEV sequences.

**Figure-5:**
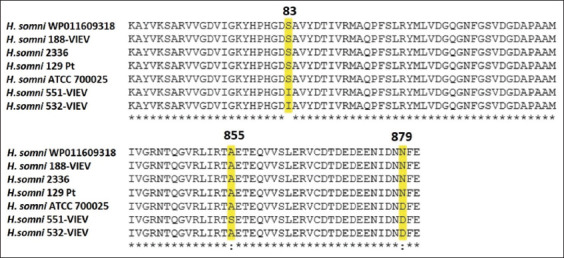
Amino acid substitutions in *Histophilus somni* topoisomerase II protein sequences.

Analysis of the DNA topoisomerase II gene (*gyrA*) in 551-VIEV and 532-VIEV revealed a substitution in codon Ser83Ile. A similar substitution important for the development of resistance to fluoroquinolones was described in *Pasteurella multocida* [24]. Resistance to fluoroquinolones corresponds to a pair of *gyrA* mutations - in positions 83 and 87 [24]. However, no substitution was found at position 87 of the *H. somni* sequences that have been studied. The Ala855Ser substitution was identified in 551-VIEV. The Asn879Asp substitution was found in three tested samples, though data on the significance of codon substitutions are lacking in the literature (Figure-5).

**Figure-6:**
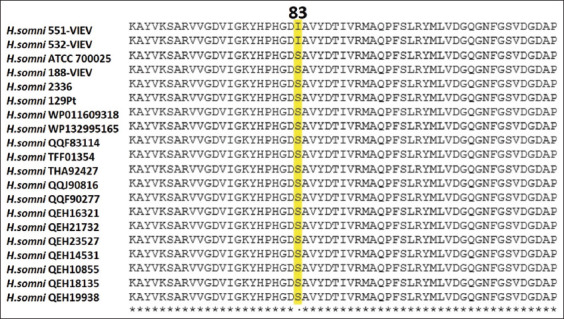
Alignment fragment of topoisomerase II protein sequences from the studied and Canadian *Histophilus somni* samples. The amino acid substitution at position 83 is shown.

Interestingly, resistant *H. somni* strains 551-VIEV and 532-VIEV were cultured from the bovine semen imported from Canada. Analysis of the *gyrA* sequences of *H. somni* isolated in 2015 in Canada and submitted to the NCBI found no such substitution at position 83 (Figure-6). It may be assumed that in recent years the fluoroquinolones resistance has increased in the *H. somni* strains circulating in Canada.

According to the literature, substitutions at positions 80 and 84 in the topoisomerase IV (*parC*) gene were found to be significant for resistance to fluoroquinolones [25]. These codon substitutions were not detected in all our sequences that have been analyzed.

## Conclusion

This article describes the features of the complete genomes of the three strains of *H. somni* isolated in Russia, and the genome of the *H. somni* ATCC 700025 strain used as a control in assessing the growth-promoting properties of nutrient media, as well as in checking the specificity of diagnostic kits intended for biochemical identification and determination of metal and antibiotic resistance of bacteria [26]. Complete genomes data can be used for the selection of candidate strains for vaccine development and to study *H. somni* pathogenicity. Antibiotic resistance genes have been identified in the *H. somni* samples isolated from the bovine semen provided by the Canadian Breeding Center. Further studies of *H. somni*, isolated from both animal material affected by pathogens and cryopreserved bovine semen used for artificial insemination are needed to control the risk of spreading antibiotic-resistant or pathogenic *H. somni* strains.

## Authors’ Contributions

SY: Conceptualized and designed the study, analyzed the data, and drafted and revised the manuscript. IT: Performed genome sequencing. VG: Performed genome assembly. JP: Compared the studied genomes and analyzed the data. All authors have read and approved the final manuscript.
